# Patterns of Variation at *Ustilago maydis* Virulence Clusters 2A and 19A Largely Reflect the Demographic History of Its Populations

**DOI:** 10.1371/journal.pone.0098837

**Published:** 2014-06-02

**Authors:** Ronny Kellner, Christian Hanschke, Dominik Begerow

**Affiliations:** 1 MPRG Fungal Biodiversity, Max Planck Institute for Terrestrial Microbiology, Marburg, Germany; 2 Department of Geobotany, Ruhr-Universität Bochum, Bochum, Germany; Universidade de Sao Paulo, Brazil

## Abstract

The maintenance of an intimate interaction between plant-biotrophic fungi and their hosts over evolutionary times involves strong selection and adaptative evolution of virulence-related genes. The highly specialised maize pathogen *Ustilago maydis* is assigned with a high evolutionary capability to overcome host resistances due to its high rates of sexual recombination, large population sizes and long distance dispersal. Unlike most studied fungus-plant interactions, the *U. maydis – Zea mays* pathosystem lacks a typical gene-for-gene interaction. It exerts a large set of secreted fungal virulence factors that are mostly organised in gene clusters. Their contribution to virulence has been experimentally demonstrated but their genetic diversity within *U. maydis* remains poorly understood. Here, we report on the intraspecific diversity of 34 potential virulence factor genes of *U. maydis*. We analysed their sequence polymorphisms in 17 isolates of *U. maydis* from Europe, North and Latin America. We focused on gene cluster 2A, associated with virulence attenuation, cluster 19A that is crucial for virulence, and the cluster-independent effector gene *pep1*. Although higher compared to four house-keeping genes, the overall levels of intraspecific genetic variation of virulence clusters 2A and 19A, and *pep1* are remarkably low and commensurate to the levels of 14 studied non-virulence genes. In addition, each gene is present in all studied isolates and synteny in cluster 2A is conserved. Furthermore, 7 out of 34 virulence genes contain either no polymorphisms or only synonymous substitutions among all isolates. However, genetic variation of clusters 2A and 19A each resolve the large scale population structure of *U. maydis* indicating subpopulations with decreased gene flow. Hence, the genetic diversity of these virulence-related genes largely reflect the demographic history of *U. maydis* populations.

## Introduction

Fungal plant parasites exhibit a large repertoire of biotrophic interactions. Domestication and practices of modern agro-ecosystems can enforce iterative bottlenecks and expansions on host and pathogen populations such that the genetic structure of extant pathogen populations more strongly reflect demographic than adaptive processes [1], [2]. The fungal pathogen *Ustilago maydis* establishes an intimate biotrophic interaction with its domesticated host plant *Zea mays* (maize) [3], [4]. As shown by population studies, agro-ecosystem-dependent bottlenecks and radiations dramatically imprinted the genetic structure of *U. maydis* such that the extant intraspecific variation reflects mainly evolution since the time of early domestication [5]. Studies based on amplified fragment length polymorphism fingerprinting and simple sequence repeat analyses demonstrated low gene flow among major populations of *U. maydis* from North and South America [5], [6]. This indicated the majority of genetic variation within *U. maydis* accumulated in the last ∼10,500 years. During domestication maize was successively transformed from its progenitor teosinte into more than 2,000 known genetic varieties, which include modern maize inbred lines [7], [8]. Domestication traits of maize underwent a reduction of diversity, and population size dramatically increased, especially in the last 200 years [9]–[11]. However, a considerably high level (∼70%) of genetic variation was retained in current maize lines. [11]. Studies on modern maize varieties demonstrated that quantitative resistance against *U. maydis* involves several quantitative trait loci (QTL), which each contribute to the frequency and severity of infection [12].

*Ustilago maydis* originated as a pathogen on teosinte and parasitises all maize varieties along with their wild progenitor in agricultural and wild populations. Owing to the lack of a gene-for-gene interaction many gene products quantitatively contribute to the virulence of *U. maydis*. As shown by ultrastructural studies, the interaction in this pathosystem is based on a characteristic vesicular system [13], [14] that highlights the important role of fungal secreted effector proteins. Effectors secreted into the apoplast by *U. maydis* are supposed to cross the plant cell membrane [3], [15] and to interfere with host defenses, similar to other fungal parasites [16]. A large portion of the transcriptome of *U. maydis* is predicted to entail secreted proteins, approximately 426 out of 6786 genes. These include, for example, the “maize induced genes” (*mig*) [17]. However, this number may be even higher when modes of non-conventional secretion are considered [4], [18]. Remarkably, many secreted protein-encoding genes of *U. maydis* are organised in gene clusters that are co-regulated and induced during pathogenesis. Moreover, deletion strains that lacked individual gene clusters exhibited altered virulence on the maize variety ‘Early Golden Bantam’ [3], [4].

Several virulence gene clusters of *U. maydis* have homologs in the related smut fungus *Sporisorium reilianum*. This provides the opportunity to assess evolutionary patterns in homologous genome regions as well as homologous genes. Comparative genomics revealed that *U. maydis* and *S. reilianum* have largely syntenic genomes and high sequence identities of homologous genes [19]. However, virulence gene clusters and secreted protein-encoding genes are enriched in genomic regions of very low sequence identity [19]. Hitherto, little is known about the role of these so-called divergence clusters but the accumulation of potential virulence genes in rapidly evolving genomic regions suggests that they promote adaptive processes in the molecular “arms race” of host resistance and fungal virulence. In particular, the impact of demographic processes on virulence-related genes of *U. maydis* within these regions is poorly understood.

To gain first insights into the intraspecific genetic diversity of virulence in *U. maydis*, we examined a selection of virulence-related genes from single isolates across larger geographic distances. We analysed genetic polymorphisms in 34 potential virulence factor genes of *U. maydis* in 17 isolates from Europe, North America and Latin America. We focused on gene cluster 2A, which is associated with virulence attenuation; cluster 19A, which is crucial for virulence, and the cluster independent effector gene, *pep1* [4], [20]. These were compared to 14 putative non-virulence genes and 4 house-keeping genes. Low levels of genetic diversity were revealed in the putative virulence factor genes across large geographic distances. In addition, coalescence analyses of virulence clusters 2A and 19A indicated a subpopulation structure with limited gene flow between isolates of geographically distinct regions. This was indicative of strong impact from demographic processes on these virulence gene clusters.

## Results

### Gene content and single nucleotide polymorphisms

In order to study polymorphisms of virulence-associated genes we completely or partially sequenced 48 genes from up to 17 isolates of *U. maydis* that represented geographical variation from Europe, North America and Latin America (Tables 1, S1, S2). Of these 48 genes, 34 encoded putatively secreted proteins and 14 genes encoded putatively non-secreted proteins. In particular, the entire virulence cluster 2A was sequenced spanning 20,725 basepairs (bp) from the left cluster-flanking gene *um01233* to the right cluster-flanking gene *um01242* [16]. We partially sequenced 37 genes from virulence cluster 19A and its flanking regions. Of these 37 genes, 25 encoded putatively secreted proteins of cluster 19A and 12 genes encoded putatively non-secreted proteins. Gene-specific primer pairs were designed for amplification of a 267 to 645 bp region of the respective gene (Table S3). The cluster 19A dataset of each strain comprised 15,613 bp, which represents 62% and 18% of the sequences of genes encoding secreted and non-secreted proteins, respectively. The entire virulence gene *pep1* (*um01987*) was sequenced using primers directed against non-coding cis- and trans-flanking regions of *pep1*. For comparison, we sequenced parts of the house-keeping genes *ef1-α* (*um00924*), *gapdh* (*um02491*), *rpb1* (*um03863*) and ITS containing *5.8S* rDNA, encoding elongation factor 1-alpha, glyceraldehyde 3-phosphate dehydrogenase, RNA polymerase II subunit 1, and the internal transcribed spacer containing *5.8S* rDNA, respectively (Table 1). Details of strains, amplicons and primers are given in Tables S1, S2 and S3. Sequences have been submitted to GenBank with the accession numbers JF313619-JF313649 and HQ002861-HQ003216 listed in Table S4.

**Table 1:** Summary of virulence and house-keeping genes sequence data.

Parameters	*pep1*	Cluster 2A secreted	Cluster 19A secreted	Cluster 19A non-secreted	ITS1/2 + *5.8S*	*gapdh*	*ef1-α*	*rpb1*
Δ phenotype	non-pathogenic	hypervirulent	reduced virulence	NA	lethal	NA	NA	lethal
N	17	13	10	10	15	16	17	17
sequenced length in bp	537 (537)	20725 (10026)	10624 (15291)	5423 (25617)	703	864 (1433)	969 (1380)	624 (5325)
haplotypes	5	12	8	7	1	5	3	1
π (JC) x 10^−3^	3.96	2.53 (4.16)	2.72	2.54	0	1.87	0.68	0
SNP/kb	11.17	7.18 (11.10)	6.97	4.61	0	5.78	3.10	0

Δ phenotype, knockout phenotype (*pep1*: [20]; cluster 2A and cluster 19A: [4]; N, number of isolates analysed; π, nucleotide diversity based on the average number of nucleotide differences between two random sequences; brackets enclose sequence lengths and values of complete coding sequences; NA, not available.

The applied sequencing approach revealed that each gene was present in all investigated strains (Tables S1, S2). Furthermore, sequences of the entire cluster 2A revealed a conserved gene content and gene order in all strains. In total, we obtained 282 polymorphic sites in 41 out of 52 genes. In particular, 102, 89 and 91 single nucleotide polymorphisms (SNPs) represent non-synonymous, synonymous and unique polymorphisms, respectively (Table 1). Each observed SNP consists of two alleles, and each polymorphic codon is based on one SNP except for one polymorphic amino acid in the gene *um05301* of the European strain RK124, which is based on two SNPs.

### Nucleotide diversity of house-keeping genes and genes encoding secreted and non-secreted proteins

**Figure 1:**
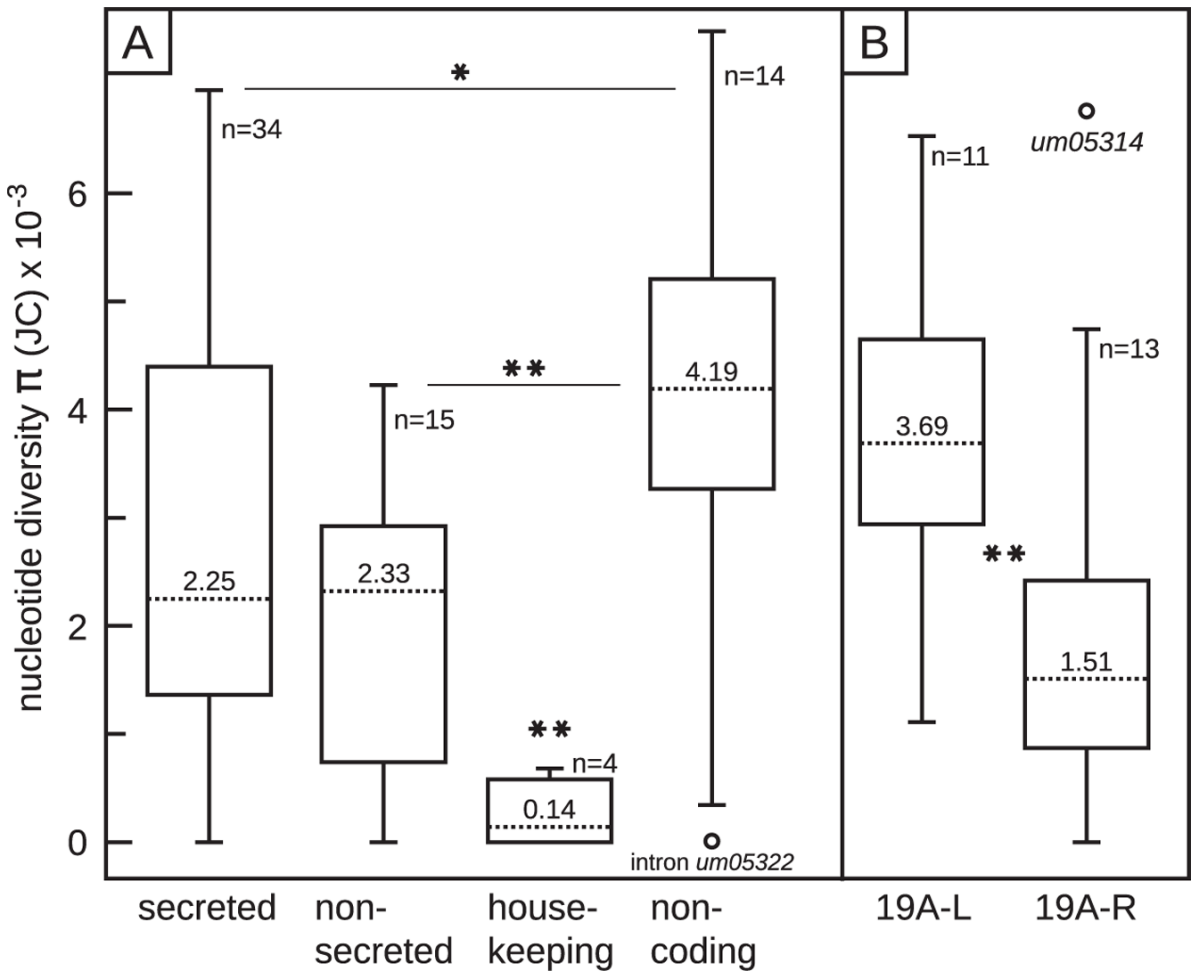
Boxplot comparisons of nucleotide diversity π datasets. (A) Nucleotide diversity boxplot diagrams of putatively secreted and non-secreted protein-coding as well as house-keeping genes (*ef1-α*, *rpb1*, *gapdh* and ITS including *5.8S* rDNA) and non-coding regions. (B) Nucleotide diversity boxplot diagrams of sub-datasets of cluster 19A. 19A-L: left part of cluster 19A genes encoding secreted proteins (*um05294* – *um10555*); 19A-R right part of cluster 19A genes encoding secreted proteins (*um05305* – *um05319*); Dotted line: median; n: number of sequences per dataset. *: p≤0.05; **: p≤0.01 in Student's t-test.

To survey genetic variation within *U. maydis* we calculated the nucleotide diversity *π* for all genes and non-coding regions. The overall mean nucleotide diversity π of all genes of this study was 0.0032. Student's t-tests of π value datasets from cluster-associated genes including *pep1*, house-keeping genes and non-coding genes revealed significant differences in nucleotide diversity (p<0.001). Thus, house-keeping genes exhibited significantly lower π values (10-fold) and non-coding regions revealed significantly higher π values (approximately 2-fold) compared to the virulence cluster-associated genes (Figure 1A). In particular, 5.8S, non-coding ITS rDNA and *rpb1* exhibited no sequence polymorphisms and are identical to the *U. maydis* reference genome (Table 1; MUMDB). Exon sequences of *ef1-α* and *gapdh* revealed π-values of 0.00068 and 0.00028, respectively (Table 1). However, there was no significant overall difference in nucleotide diversity π between genes encoding secreted and non-secreted proteins (Figure 1A). Thus, the overall nucleotide diversity in *U. maydis* is low with significant differences between house-keeping genes and cluster-associated genes but no significant difference between secreted and non-secreted protein-encoding genes.

### Tests for natural selection

**Figure 2:**
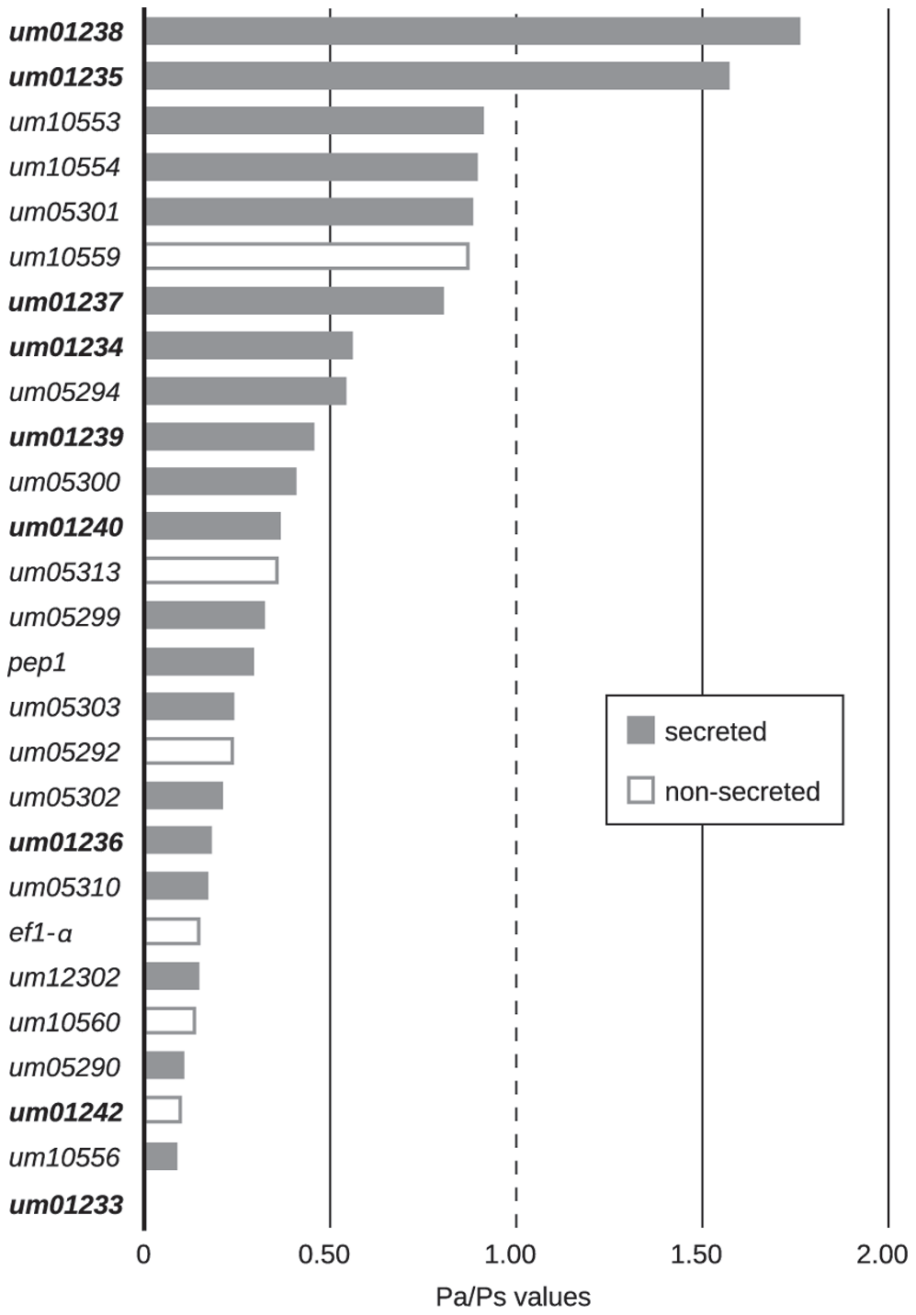
Ratios of Pa and Ps. Listed are all genes that contain non-synonymous and synonymous mutations. Grey bars indicate putatively secreted protein-coding genes and white bars indicate putatively non-secreted protein-encoding genes. Bold um gene numbers represent genes of cluster 2A. Light um gene numbers represent genes of cluster 19A.

We tested whether single virulence-related genes and non-virulence genes underwent positive selection as the nucleotide diversity π values of the investigated genes of *U. maydis* varied between 0.000 to 0.007 (Table S2). To this end, we first calculated Tajima's D, Fu & Li's D* and Fu & Li's F* summary statistics for all genes and determined their probabilities (Table S2) [21], [22]. This revealed eight genes that showed signatures of non-neutral evolution with a p-value below 0.05 for at least one of the three applied summary statistics. Of these, one gene encoded the putatively secreted effector gene *um01236* of cluster 2A, six genes encoded putatively secreted proteins of cluster 19A and one gene encoded the putatively non-secreted cluster 19A-flanking gene *um10560*. In particular, *um01236*, *um05290*, *um12302*, *um05303* and *um10560* revealed positive summary statistics that indicated a significant excess of intermediate frequency mutations. Furthermore, *um05301*, *um10556* and *um05310* revealed negative values that indicated an excess of rare mutation. To further evaluate the summary statistics and to analyse signatures of selection at the amino acid level we calculated the ratio of non-synonymous polymorphisms per non-synonymous site (Pa) to the number of synonymous polymorphisms per synonymous site (Ps). From 52 analysed genes only 27 exhibited both, non-synonymous and synonymous polymorphisms, and could be used for comparison. Of these, 20 encoded secreted proteins, 6 encoded non-secreted proteins and 1 gene encoded the house-keeping gene *ef1-α* (Figure 2, Table S5). To identify genes under natural selection, we applied a Z-test [23] that compared the rates of Pa (proportion of non-synonymous polymorphisms) and Ps (proportion of synonymous polymorphisms). Due to the low levels of genetic variation the Z-tests revealed no significant evidence for non-neutral or neutral evolution (Figure 2, Table S6). In summary, nucleotide-based tests for selection identified signatures of non-neutral evolution for eight genes of virulence clusters 2A and 19A. However, they were not confirmed by codon-based Pa/Ps statistics.

### Phylogeographic structure

**Figure 3:**
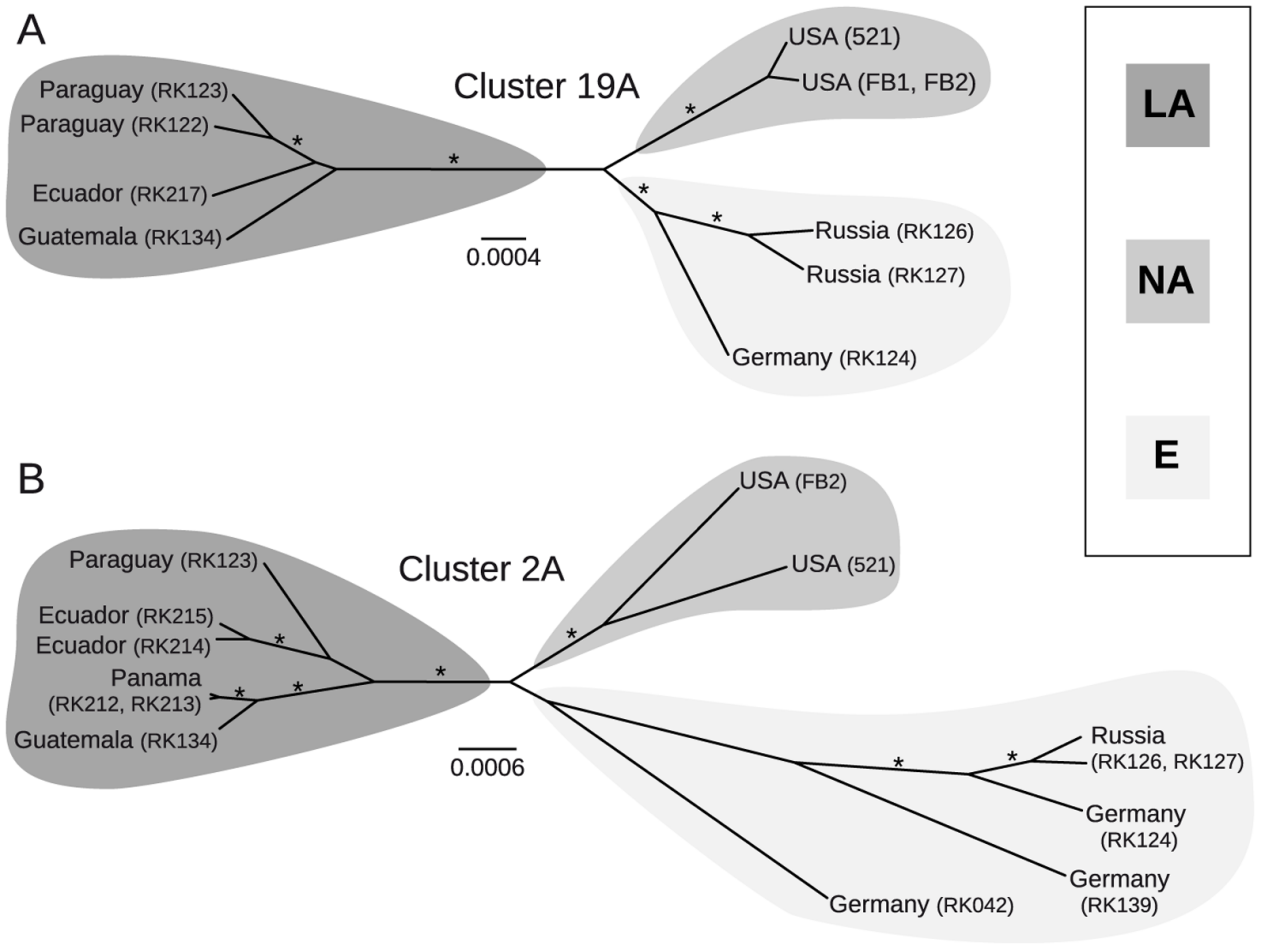
Coalescence genealogies of cluster 19A and cluster 2A sequence datasets. Coalescence-based genealogies (Beast v1.6.1) of datasets comprising (A) partial sequences of cluster 19A and (B) entire cluster 2A. Asterisks above branches correspond to a posteriori probabilities higher than 0.98. Branch lengths correspond to substitutions per site.

Population genetic analyses of *U. maydis* demonstrated genetic differentiation between geographically distinct populations and suggested the presence of genetic isolation between major populations during recent evolutionary time [5], [6]. To evaluate the genetic divergence in our dataset we first conducted separate Maximum Likelihood (ML) analyses of cluster 2A and 19A alignments. ML recovered two, strongly-supported clades, one that contained all Latin American strains (LA) and a second with all North American and European strains (NA-E; Figure S1A, B). RAxML does not account for intragenic recombination, so separate coalescence analyses were performed for both alignments. These analyses recovered the same clades from the ML analyses (Figure 3A, B). The dataset of cluster 19A favoured a third group of all European strains (E; Figure 3B), which was supported with a weak *posteriori* probability for the dataset of cluster 2A (Figure 3A). Analyses of sub-datasets of genes encoding either secreted or non-secreted proteins revealed the same clades with lower support values (data not shown). Based on these results we concluded the presence of subpopulations in terms of decreased gene flow between individuals from LA, NA and E.

### Intraspecific diversity of cluster 19A gene sequences

**Figure 4:**
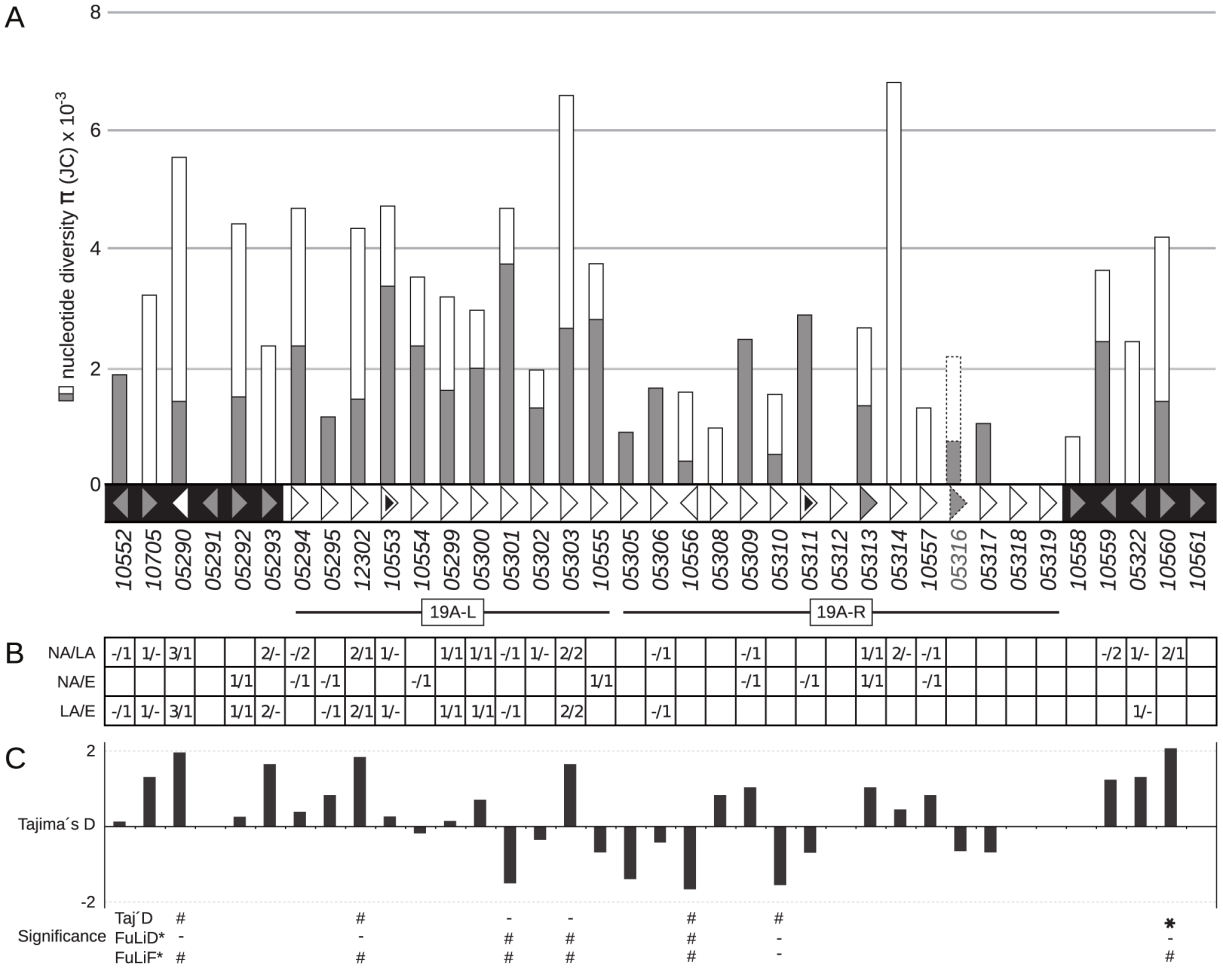
Cluster 19A nucleotide diversity π, fixed polymorphisms and neutrality tests. (A) Genes are illustrated in the genomic gene order. Arrowheads below the graph indicate their reading direction. White and grey arrows depict secreted and non-secreted protein-coding genes, respectively. Black sections are cluster-flanking regions. Black triangles highlight genes that revealed truncated alleles. Numbers correspond to *U. maydis* gene numbers in (MUMDB). Two-section columns height corresponds to nucleotide diversity π with Jukes-Cantor (JC) correction (scaled on left axis) and its sections reflect the ratio of non-synonymous (grey) and synonymous (white) substitutions. The pseudogene *um05316* is dotted. (B) Number of fixed polymorphisms between subpopulations of North America (NA), Europe (E) and Latin America (LA). First and second numbers refer to non-synonymous and synonymous substitutions, respectively. (C) Tajima's D statistics for gene datasets of cluster 19A. P-values of Tajima's D (Taj'D) and corresponding Fu & Li's D* (FuLiD) and F* (FuLiF) statistics are highlighted with # (p<0.05) and * (p<0.01).

Within cluster 19A, we identified genes containing no polymorphisms (*um05312*, *um05318*, *um05319* and *um10561*), genes containing only synonymous mutations (*um10705*, *um05293*, *um05308*, *um05314*, *um10557*, *um10558* and *um05322*), genes containing only non-synonymous mutations (*um10552*, *um05295*, *um05305*, *um05306*, *um05309*, *um05311* and *um05317*) as well as genes containing both, synonymous and non-synonymous mutations (*um05290*, *um05294*, *um12302*, *um10553*, *um10554*, *um05299*, *um05300*, *um05301*, *um05302*, *um05303*, *um10555*, *um10556*, *um05310*, *um10559*, *um10560*; Figure 4A). In both Latin American strains (RK122 and RK217), one nucleotide polymorphism in *um10553* introduced a stop codon at position 173 of the encoded protein. In addition, *um05311* of RK217 encodes a stop codon at amino acid position 167 that should lead to a truncated version of the protein (Figure 4A). However, as sequence information of cluster 19A is clearly limited due to short partial sequences of single genes the power of the applied summary statistics is likely reduced.

Based on nucleotide diversity π, the genetic variation seemed to be heterogeneously distributed along cluster 19A (Figure 4A). Therefore we split the cluster in two distinctive regions, which we named 19A-L (ranging from *um05294* to *um10555*) and 19A-R (ranging from *um05305* to *um05319*). Student's t-tests revealed significant differences in nucleotide diversity between 19A-L and 19A-R (Figure 1B, p<0.01). Thus, 19A-L carries most of the cluster variation whereas 19A-R predominantly contains genes with low variation, genes that exhibit only synonymous polymorphisms and genes without polymorphisms (Figure 4A). In addition, sequences of 19A-L together with cluster 19A left flanking gene sequences are enriched in fixed differences compared to 19A-R and cluster 19A right flanking gene sequences (Figure 4B). However, this bilateral distribution of polymorphisms is not reflected by Tajima's D, Fu & Li's D* and Fu & Li's F* summary statistics. They revealed a homogeneous distribution of seven selectively non-neutral loci including the cluster-flanking genes *um10560* and *um05290* as well as the cluster 19A genes *um12302*, *um05301*, *um05303*, *um10556* and *um05310* (Figure 4C).

We tested for linkage disequilibrium (LD) between all polymorphic sites of cluster 19A as the evolutionary rate of locally linked loci can hitchhike with favorable or non-favorable mutations of neighboring loci due to recombinational linkage. The LD plot revealed a patchy distribution of LD between both close and distant sites of cluster 19A, which rejected the hypothesis of local LD in cluster 19A (Figure S2). In summary, cluster 19A displays a significant, heterogenic pattern of sequence variation that has been created by recombination.

### Intraspecific diversity along cluster 2A

**Figure 5:**
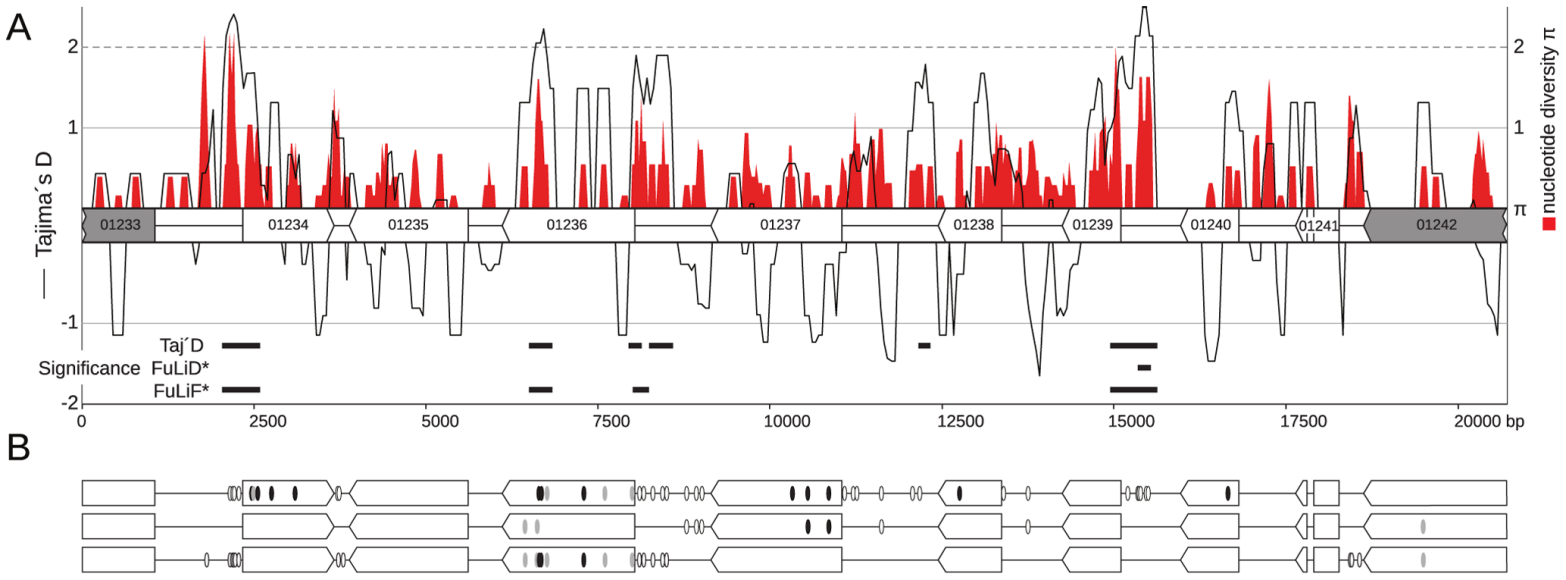
Cluster 2A nucleotide diversity π, fixed polymorphisms and neutrality tests. (A) Genes and non-coding regions are illustrated in the genomic gene order. Arrows indicate their reading direction. Numbers correspond to *U. maydis* gene numbers in [51]. Grey arrows depict genes that encode non-secreted proteins and white arrows depict genes that encode secreted proteins. The red graph illustrates sequence diversity π (x 10^−3^) of a sliding window analysis (window length: 200; step size: 50) scaled on the right axis. The black curve illustrates a sliding window analysis (window length: 200; step size: 50) of Tajima's D statistics scaled on the left axis. Bars below the graph indicate segments with p-values <0.05 for Tajima's D (Taj'D) and corresponding Fu & Li's D* (FuLiD) and F* (FuLiF) statistics. (B) Fixed differences between datasets of subpopulations North America (NA), Europe (E) and Latin America (LA). Ovals highlight positions of fixed polymorphisms in non-coding (white) and coding regions comprising non-synonymous (black) and synonymous (grey) substitutions.

In contrast to cluster 19A the genes of cluster 2A revealed no heterogenic pattern of variation as shown by a sliding window analysis (window length: 200, step size: 50; Figure 5A). However, there are some regions spanning up to 650 bp that lack polymorphisms, e.g. encoding the C terminus as well as the 3′ non-coding region of *um01240* and the C terminus of *um01242* (Figure 5A).

To test for natural selection we estimated Tajima's D, Fu & Li's D* and Fu & Li's F* summary statistics in sliding window analyses of cluster 2A sequence alignments (window size 200 bp, step size 50 bp). The null hypothesis is significantly rejected in five regions. Thereof, a 5′ non-coding region of *um01239* is supported by all three methods, the 5′non-coding regions of *um01234* and *um01236* as well as a C-terminal region of *um01236* are supported by Tajima's D and Fu & Li's F*, whereas a 3′ non-coding region of *um01238* is solely supported by Tajima's D (Figure 5A). However, codon-based tests for natural selection did not support non-neutral evolution for any gene of cluster 2A. Because coalescent analyses suggested a subpopulation structure for *U. maydis* (Figure 3) we examined whether the non-neutrally evolving regions of cluster 2A contain polymorphisms that are fixed between the two isolates from North America (NA), the five isolates from Europe (E) and the six isolates from Latin America (LA). Out of 166 polymorphic sites, 39 sites were polymorphic in a single strain. Isolates from NA, E and LA had polymorphic sites at 44, 82 and 46 respectively. In total 50 polymorphic sites (Figure 5B) constitute fixed differences that were non-polymorphic within the subpopulation but polymorphic between the subpopulations NA, E and/or LA. This includes 12 non-synonymous polymorphisms, 7 synonymous polymorphisms and 31 polymorphisms in non-coding regions. Of these, 24 fixed differences coincide with regions that revealed signatures of non-neutral selection (Figure 5C). In addition, eight fixed differences were present in a single gene (*um01236*) comprising all of its non-synonymous polymorphisms.

Hence, coding regions and non-coding regions of cluster 2A in *U. maydis* revealed signatures of non-neutral evolution. In addition, these regions are enriched with polymorphisms that seem to be fixed in geographically isolated subpopulations.

## Discussion

In molecular “arms races” host resistance and fungal virulence underlie strong selection with recurrent adaptive evolution of resistance and virulence-related genes. Consequently, virulence genes frequently show signatures of positive selection [24], [25]. However, in agro-ecosystems such genetic signatures are often obliterated due to demographic fluctuations [2]. In the present study we examined the genetic diversity of two virulence gene clusters, 2A and 19A, of the maize pathogen *U. maydis*. This demonstrated an overall low intraspecific genetic variation of these virulence-related genes across large geographic distances and across subpopulation barriers.

The nucleotide diversities π of all investigated *U. maydis* genes were remarkably low compared to the diversity levels within the fungal hemi-biotrophic ascomycete *Zymoseptoria tritici* [26]. The comparison of three genomes of *Z. tritici* documented mean π values above 0.01 for single chromosomes. In contrast, the virulence clusters 2A and 19A, the cluster-flanking genes, the house-keeping genes as well as the non-coding regions of 17 isolates of *U. maydis* revealed π values below 0.003, which indicated a loss of genetic diversity in *U. maydis*. The overall sequence divergence is too low to rely on tests for natural selection of genes or single sites [27], [28]. Furthermore, sequence alignments of the investigated virulence genes of *U. maydis* with their putative orthologs from *S. reilianum* and *U. hordei* are highly unreliable, which renders interspecific evolutionary analyses impossible [19], [29]. Hence, future grass smut analyses of fast evolving genes are in need of genome information from isolates with altered host specificities, e.g. *U. maydis* isolates from teosinte, the wild progenitor of maize, or more closely related species of *U. maydis*, e.g. *U. bouriquetii* or *U. vetiveriae* [30], [31].

In contrast to increased levels of divergence on the interspecies level between *U. maydis* and the related maize pathogen *Sporisorium reilianum* [8], our comparison within *U. maydis* revealed no significant difference between virulence-related and non-virulence genes (Figure 1). These low levels of genetic variation demonstrated that virulence clusters 2A and 19A diverged before the adaptation to modern maize. In addition, they reflect the young age of recent *U. maydis* populations [5]. If a maximum age of 10,000 years for populations of *U. maydis* is assumed substantial levels of intraspecific genetic variation were retained at numerous virulence and non-virulence loci, which likely reflects its large population sizes. In addition, we noted that the genetic diversity of virulence clusters 2A and 19A both reflect the large scale sub-population structure of *U. maydis* (Figure 4). Hence, the investigated virulence genes largely reflect the demographic history of these populations and signatures of natural selection are, if present at all, strongly imprinted. The discrepancy between the non-significant codon-based tests for natural selection and the summary statistics, e.g. Tajima's D confirm this finding as the latter tests are known to be sensitive for demographic impacts. However, only three genes of cluster 19A and two cluster 19A-flanking genes seem to be subject to purifying selection as they revealed no polymorphisms among individuals of distant geographic regions (Figure 5). For the other genes Pa/Ps tests for selection are non-significant due to low sampling depth. In conclusion, the demographic history of *U. maydis* requires deeper sampling to determine methods of natural selection.

It remains unclear whether virulence cluster genes of *U. maydis* were directly involved in the adaptation to domesticated maize. For example, the non-virulent *pep1* deletion mutant of *U. maydis* can be fully rescued by the *pep1* ortholog of the barley smut *U. hordei*, which questions a host-specific function in *U. maydis* [20]. Similarly, gene products of clusters 2A and 19A could act as general rather than host-specific virulence factors. In consequence, they would carry out conserved functions that are subject to purifying selection, hence, leading to less genetic variation. The recent functional characterization of cluster 19A provided evidence for its role in quantitative virulence (Brefort *et al*. 2014, in press in PLoS Pathogens). Although significantly impaired in virulence cluster 19A, deletion mutants were able to complete their life cycle with the production of few spores. Cluster 19A sub-deletion experiments identified 4 individual genes and one gene family that independently contributed to virulence or anthocyanin production. Interestingly, deletion of the left part of cluster 19A led to a dramatic reduction of spore production and the loss of anthocyanin production (Brefort *et al*. 2014, in press in PLoS Pathogens) whereas the right part of cluster 19A was only weekly attenuated in virulence. Our study revealed that the left part including *um05294* to *um19554* (*tin1-1* to *tin1-5*) and *um05302* (*tin2*) contains significantly more polymorphisms compared to the right part of cluster 19A (Figures 1B, 4). This indicates that this region might undergo diversification in *U. maydis*. However, a correlation of these virulence phenotypes and our patterns of intraspecific genetic variation is problematic because single genotypes of *U. maydis* can show dramatic differences in virulence on different maize varieties (Kellner and Begerow unpublished data). Additionally, the maize variety Early Golden Bantam, which is mainly used in functional analyses, likely does not represent the host genotype that the ancestors of investigated isolates of *U. maydis* evolved on.

Domestication selects for specific phenotypes and involves the fixation of specific alleles. In crops these selective sweeps largely acted on regulatory elements of domestication trait-related genes involving altered transcriptional activity [32]. Similarly, the 5′non-coding regions of *um01234*, *um01236* and *um01239* of cluster 2A revealed weak evidence for non-neutral evolution. In case of non-neutral evolution these regions could play a role in transcriptional or post-transcriptional control indicating the relevance of altered gene expression or altered mRNA stability for the evolution of virulence in *U. maydis* (Figure 5). Similar to RNA-binding proteins of *U. maydis*, e.g. Khd4 and Rrm4 that regulate their targets by binding to specific sequences in 5′ and 3′ untranslated regions (UTRs) [33], SNPs within UTRs of virulence genes could alter gene expression and stability. A linkage of altered gene expression and adaptive evolution is proclaimed by several studies. In *Saccharomyces cerevisiae* experimental evolution altered gene expression levels of several hundred genes within 250 asexual generations and resulted in genotypes that were better adapted to glucose limitation and produced less ethanol [34]. In a recent study, the transcription factor Efg1p of *Candida albicans* revealed a linkage of expression levels and immune status of the host [35]. Hence, the intraspecific sequence variation in 5' non-coding regions of *U. maydis* virulence cluster genes could reflect recent adaptive processes in gene regulation although the linkage of sequence polymorphism and altered gene expression remains to be experimentally verified and evaluated.

In summary, the overall low levels of genetic variation of virulence clusters 2A and 19A across large geographic distances and subpopulations reflect the demographic history of *U. maydis* populations. Future comparative genomic studies of *U. maydis* are well advised to consider more in depth sampling compared to other fungal parasite model species.

## Materials and Methods

### Fungal isolates, primer design and sequencing

Isolates of *Ustilago maydis* used in this study were isolated from herbarium material originating from Europe, North America and South America. In total, 18 isolates representing 11 different localities were isolated and grown as described in [31] (Table S1). The isolate collection contained two isolates from teosinte (*Zea mays ssp. parviglumis*), the wild progenitor of maize. Genomic DNA of haploid *U. maydis* cultures was isolated by the method of [36]. Primers for *pep1*, cluster 2A and cluster 19A were designed based on the genome sequence of *U. maydis* (MUMDB). Primer properties were evaluated with OligoCalc [37], [38] or Clonemanager v9.0 (Sci-Ed Software, Cary). ITS rDNA containing *5.8S* was amplified using the primers ITS1 and ITS4 [39]. *rpb1* was amplified using the primers RoK157 and RoK158 [31]. *ef1-α* was amplified using primers 983F and 2018R, and sequenced with primers 1567R and 2018R [40]. *gapdh* was amplified with primers G3PD-581F and G3PD-2020R, and sequenced with these external primers and the internal primer G3PD-1501R [41]. Detailed primer descriptions are given in Table S3.

For DNA amplification ≤5 kb, Phusion High-Fidelity Polymerase (Finnzymes, Espoo) and for amplification >5 kb, KOD Xtreme Polymerase (Merck Biosciences, Nottingham) were used following manufacturer's instructions. PCR products were purified directly or through gel purification using my-Budget Double Pure Kit (Bio-Budget, Krefeld). The complete gene *pep1* (537 bp) was sequenced from 16 isolates. The entire cluster 2A was amplified from twelve isolates spanning 20,725 bp from the left flanking gene *um01233* to the right flanking gene *um01242*. Sequencing of cluster 2A fragments was done by LGC Genomics (Berlin) applying 454 and shot gun sequencing. 26 genes of cluster 19A and 11 of its flanking genes were partially sequenced in nine isolates. PCR fragments were sequenced on an ABI 3130XL Genetic Analyser (Applied Biosystems) from the sequencing service of the Biochemistry Department at the Ruhr-Universität Bochum. Nucleotide sequences of *pep1*, *gapdh*, *ef1-α*, cluster 2A and cluster 19A genes have been deposited in GenBank under the accession numbers listed in Table S4.

### Sequence alignment, gene identification and domain prediction

Obtained sequences were quality checked and edited by hand if needed using Sequencher 5.0 (Gene Codes Corporation, Ann Arbor). For phylogenetic and coalescence analyses, sequences were aligned with MAFFT 6.707 applying default settings and a maximum number of 1,000 iterations. Subsequently, leading and trailing gaps were manually removed from the alignment. Genes, exons, introns and intergenic regions within respective regions were identified by comparison with sequences of the *U. maydis* genome using the genome browser (MUMDB). In order to evaluate correlations of gene function and substitution patterns we adopted information from MUMDB and additively predicted functional domains applying SMART blast searches [42], [43]. For comparison with *S. reilianum* we used additional information from the genome browser [44]. Information on synteny conservation and genetic homology between *U. maydis* and *S. reilianum* were obtained from MUMDB.

### Phylogenetic and coalescence analyses

Maximum Likelihood (ML) analyses were performed with RAxML 7.0.4 [45], [46]. RAxML 7.0.4 conducted 1000 bootstrap replicates using a rapid bootstrap algorithm applying GTRMIX approximation [47]. In the subsequent ML search for the best scoring ML tree starting from each 5^th^ bootstrap tree the more accurate GTRCAT approximation was applied. Bootstrap support values were mapped on the most likely tree that was visualised and edited in FigTree v1.3.1. For the analysis of cluster 19A, sequences were concatenated. For each partition, RAxML estimated and optimised individual α-shape parameters, GTR-rates and empirical base frequencies.

The Bayesian Markov Chain Monte Carlo (MCMC) method implemented in Beast v1.6.1 was used to estimate coalescence-based genealogies of cluster 19A and 2A datasets [48]. Coalescent-based gene genealogy of a combined analysis of all partial sequences of cluster 19A and its flanking genes was conducted applying the GTR+I+G model. For each partition Beast estimated individual base frequencies, α-shape parameters and GTR-rates. The entire cluster 2A was analysed applying the same settings as used for cluster 19A to each gene and each non-coding locus individually. To consider different demographic histories of *U. maydis* we, furthermore, applied and compared three different tree prior settings (constant size, exponential growth and expansion growth) in the coalescence analysis.

### DNA polymorphism and analyses of linkage disequilibrium

Identification of single nucleotide polymorphisms (SNP), synonymous and non-synonymous mutations as well as further statistics of DNA polymorphism, nucleotide diversity π, summary statistics (Tajima's D, Fu & Li's D* and Fu & Li's F* [21], [22]), ratios of Pa and Ps were conducted using DnaSP v5.10.01 [49]. For datasets of cluster 2A sliding window analyses (window length: 200, step size: 50) were performed. To analyse the differences in nucleotide diversity between datasets, we created boxplot diagrams [50] and tested for significant differences between datasets applying student's t-tests in R (www-R-project.org). In order to calculate mean π values of cluster 2A and 19A, respective loci were concatenated. Pairwise linkage disequilibrium was analysed at all polymorphic sites between all nucleotide variants. Sites containing alignment gaps, or polymorphic sites segregating for three or four nucleotides, were excluded from the analysis. Polymorphic sites with statistical significance for linkage disequilibrium (p<0.05 in two tailed Fisher's exact test and chi-square test) were plotted along the sequence using standard packages in R (www-R-project.org).

## Supporting Information

Figure S1 **Phylogeny of cluster 19A and cluster 2A.** Maximum Likelihood phylogeny (RAxML 7.0.4) of sequence alignments of cluster 19A and cluster 2A from U. maydis. From cluster 19A partial coding sequences of 37 genes and from cluster 2A complete coding sequences of 8 genes were analysed. Bootstrap values of 1000 replicates are given next to branches (asterisks indicate values ≥93). Branch lengths are scaled with respect to the expected number of nucleotide substitutions per site.
(TIF)

Figure S2 **Linkage disequilibrium dot plot cluster 19A.** Test for linkage disequilibrium between nucleotide variants at all different polymorphic sites along cluster 19A. Sequences of cluster 19A are stringed together in genomic order. Sites containing alignment gaps, or polymorphic sites segregating for three or four nucleotides, are excluded from the analysis. Plotted are polymorphic sites with statistical significance for linkage disequilibrium (p<0.05 in two tailed Fisher's exact test and chi-square test). 19A-L and 19A-R represent the sub-clusters of Figure 4.
(TIF)

Table S1 ***U. maydis*** **strain collection and amplicon summary.** Zmm: *Zea mays spp. mays*; Zmp: *Zea mays ssp. parviglumis*; Pop: amount of populations sampled; p.com.: personal communication; nd: not determined.
(DOC)

Table S2 **Summary of sequence analyses.** N: number of analysed *U. maydis* strains; haplotypes: number of haplotypes in the dataset; length: sequence length of complete genes in bp; sequenced: number of sequenced basepairs; SNPs: single nucleotide polymorphisms; nonsyn: non-synonymous mutations; syn: synonymous mutations; π (JC): nucleotide diversity calculated with DnaSP v4.0 applying the Jukes Cantor correction; TajD: Tajima's D summary statistics and corresponding probability value (SigTajD); FuLiD*: Fu & Li's D* summary statistics and corresponding probability value (SigLiD*); FuLiF*: Fu & Li's F* summary statistics and corresponding probability value (SigF*); #: p-value <0.05, *: p-value <0.01; NA: not available; NS: not significant.
(XLS)

Table S3 **Primers used in this study.**
(DOC)

Table S4 **Accession numbers.**
(DOC)

Table S5 **Rates of Pa and Ps.** Rates of Pa and Ps were calculated according to the number of non-synonymous and synonymous sites and non-synonymous and synonymous mutations per locus. stdv: standard deviation.
(DOC)

Table S6 **Z-test statistics on rates of Pa and Ps.** Codon-based test for neutral evolution averaged over all sequence pairs. The probability of rejecting the null hypothesis of strict-neutrality (Pa  =  Ps), positive selection (Pa > Ps) and purifying selection (Pa < Ps) is shown. Values of p less than 0.05 are considered significant at the 5% level. The test statistic (Pa - Ps) is shown in the Stat column. Pa and Ps are the numbers of synonymous and non-synonymous polymorphisms per site, respectively. The variance of the difference was computed using the bootstrap method (1,000 replicates). Analyses were conducted using the Nei-Gojobori method [52]. The analysis involved 13 and 10 coding sequences of cluster 2A and 19A, respectively. All positions with less than 95% site coverage were eliminated. Total amount of positions as amino acids in the final dataset is given in column “length in aa”. Evolutionary analyses were conducted in MEGA5 [53].
(XLS)
