# Supplementary figures and images for: Patterns of Variation at Ustilago maydis Virulence Clusters 2A and 19A Largely Reflect the Demographic History of Its Populations
Source: PLoS One. 2014 Jun 2;9(6):e98837. doi: 10.1371/journal.pone.0098837 (PMC4041787; doi:10.1371/journal.pone.0098837)

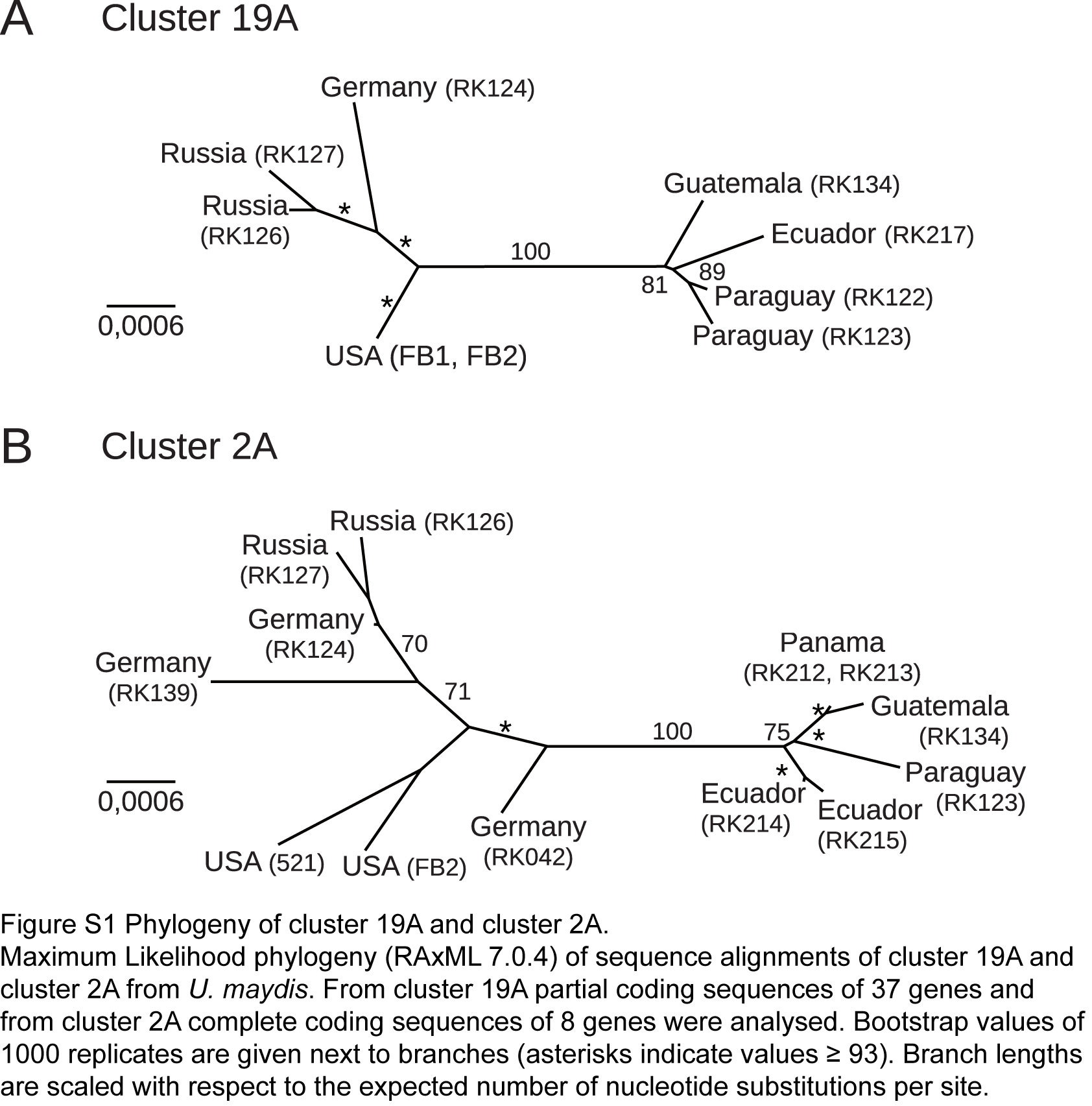

Supplement: Figure S1 — Phylogeny of cluster 19A and cluster 2A. Maximum Likelihood phylogeny (RAxML 7.0.4) of sequence alignments of cluster 19A and cluster 2A from U. maydis. From cluster 19A partial coding sequences of 37 genes and from cluster 2A complete coding sequences of 8 genes were analysed. Bootstrap values of 1000 replicates are given next to branches (asterisks indicate values ≥93). Branch lengths are scaled with respect to the expected number of nucleotide substitutions per site. (TIF) [file pone.0098837.s001.tif]

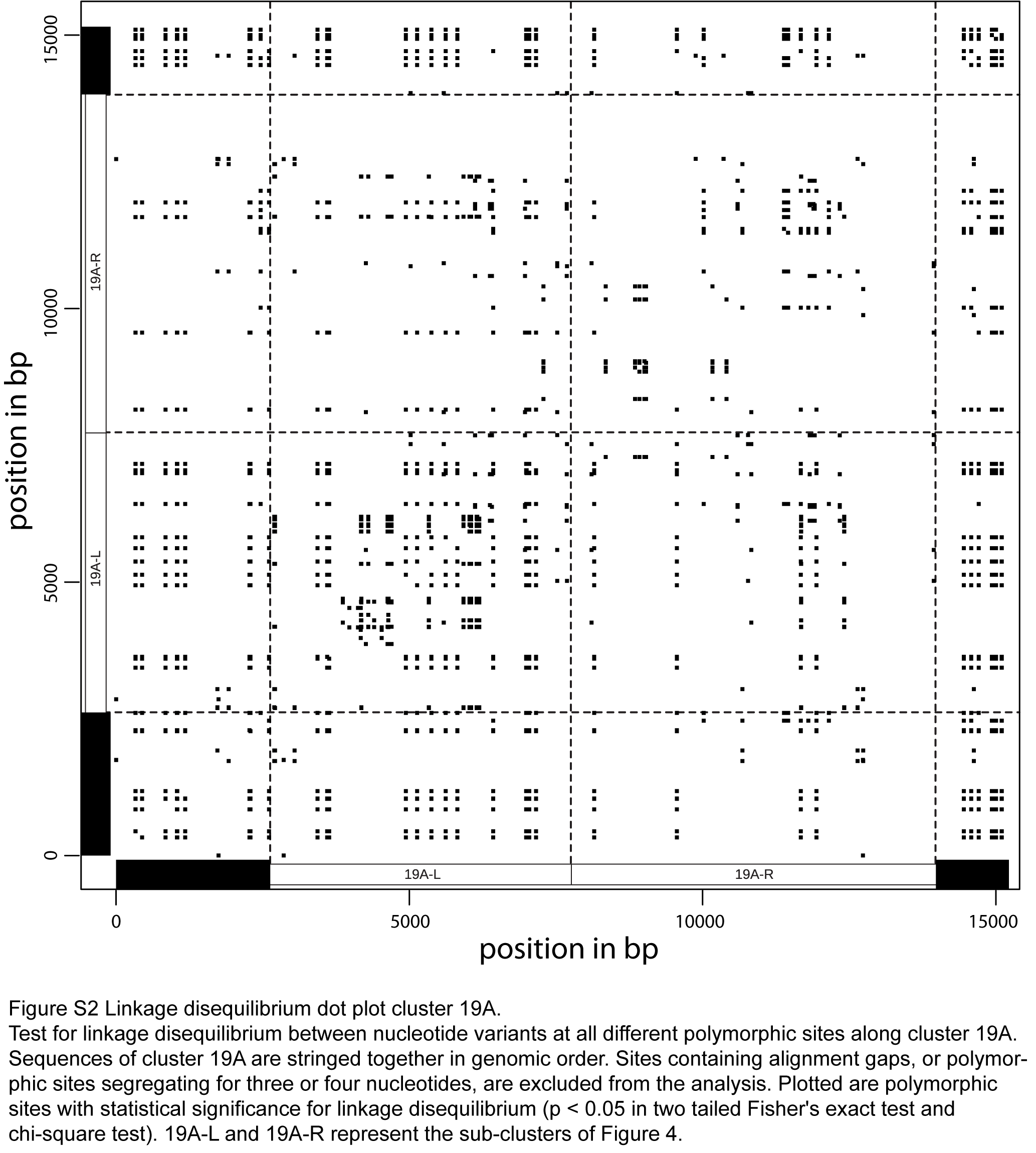

Supplement: Figure S2 — Linkage disequilibrium dot plot cluster 19A. Test for linkage disequilibrium between nucleotide variants at all different polymorphic sites along cluster 19A. Sequences of cluster 19A are stringed together in genomic order. Sites containing alignment gaps, or polymorphic sites segregating for three or four nucleotides, are excluded from the analysis. Plotted are polymorphic sites with statistical significance for linkage disequilibrium (p<0.05 in two tailed Fisher's exact test and chi-square test). 19A-L and 19A-R represent the sub-clusters of Figure 4. (TIF) [file pone.0098837.s002.tif]
