# Supplementary material for: Patterns of Variation at Ustilago maydis Virulence Clusters 2A and 19A Largely Reflect the Demographic History of Its Populations
Source: PLoS One. 2014 Jun 2;9(6):e98837. doi: 10.1371/journal.pone.0098837 (PMC4041787; doi:10.1371/journal.pone.0098837)
Supplement: Table S1 — U. maydis strain collection and amplicon summary. Zmm: Zea mays spp. mays; Zmp: Zea mays ssp. parviglumis; Pop: amount of populations sampled; p.com.: personal communication; nd: not determined. (DOC) [file pone.0098837.s003.doc]

Kellner et al. Table S1

| Strain | Origin | Host | Reference (strain designation) | pep1 | Cluster 2A | Cluster 19A | rpb1 | gapdh | ef1-α | ITS1/2 + 5.8S | Pop |
| --- | --- | --- | --- | --- | --- | --- | --- | --- | --- | --- | --- |
| FB1 | USA, Minnesota | Zmm | Banuett & Herskowitz, 1989 | + | + | + | + | + | + | + | 11 |
| FB2 | USA, Minnesota | Zmm | Banuett & Herskowitz, 1989 | + | + | + | + | + | + | + | 11 |
| RK001 | Germany, Tübingen | Zmm |  | + | nd | nd | + | + | + | + | 1 |
| RK020 | Germany, Putzbrunn | Zmm |  | + | nd | nd | + | + | + | + | 2 |
| RK042 | Germany, Tübingen | Zmm | J. P. Sampaio, p.com. (ZP399) | + | + | nd | + | nd | + | nd | 3 |
| RK122 | Paraguay | Zmm | R. Kahmann, p.com. (2107) | + | + | + | + | + | + | + | 4 |
| RK123 | Paraguay | Zmm | R. Kahmann, p.com. (2111) | + | + | + | + | + | + | + | 4 |
| RK124 | Germany | Zmm | R. Kahmann, p.com. (768) | + | + | + | + | + | + | + | 5 |
| RK126 | Russia | Zmm | R. Kahmann, p.com. (2071) | + | + | + | + | + | + | + | 6 |
| RK127 | Russia | Zmm | R. Kahmann, p.com. | + | nd | + | + | + | + | + | 6 |
| RK134 | Guatemala | Zmm | Basse et al., 2002  (CBG12-5) | + | + | + | + | + | + | + | 7 |
| RK139 | Germany, Usedom | Zmm | R. Kahmann, p.com. | + | nd | nd | + | + | + | + | 8 |
| RK212 | Panama | Zmm | J. Schirawski, p.com. (JS 179) | + | + | nd | + | + | + | + | 9 |
| RK213 | Panama | Zmm | J. Schirawski, p.com. (JS 183) | + | + | nd | + | + | + | + | 9 |
| RK214 | Ecuador, Loja | Zmp |  | + | + | nd | + | + | + | nd | 10 |
| RK215 | Ecuador, Loja | Zmp |  | + | + | nd | + | + | + | + | 10 |
| RK216 | Ecuador, Loja | Zmp |  | nd | nd | nd | nd | nd | nd | nd | 10 |
| RK217 | Ecuador, Loja | Zmp |  | nd | nd | + | nd | nd | nd | + | 10 |

Zmm: *Zea mays spp. mays*; Zmp: *Zea mays ssp. parviglumis*; Pop: amount of populations sampled; p.com.: personal communication; nd: not determined.
