# Supplementary material for: Patterns of Variation at Ustilago maydis Virulence Clusters 2A and 19A Largely Reflect the Demographic History of Its Populations
Source: PLoS One. 2014 Jun 2;9(6):e98837. doi: 10.1371/journal.pone.0098837 (PMC4041787; doi:10.1371/journal.pone.0098837)
Supplement: Table S3 — Primers used in this study. (DOC) [file pone.0098837.s005.doc]

Kellner et al. Table S3

| Primer Name | Sequence | Amplification (localisation) | Specificity |
| --- | --- | --- | --- |
| CH1 | GGT CAG GCA GGT AAC CAG AT | cluster 19A (um10558) | U. maydis |
| CH2 | CAC CAG CTG GTG GAA CGA CA |  | U. maydis |
| CH3 | CGC GCT ACC AAG GTG TTG AC | cluster 19A (um05293) | U. maydis |
| CH4 | CCC ATG AGG ATG AGC ACC AG |  | U. maydis |
| CH5 | CAG GTC CAA GCA GCT AAA GA | cluster 19A (um10556) | U. maydis |
| CH6 | CCT CCA GAG CGG ATC CTA TA |  | U. maydis |
| CH9 | CTT CAG TCC TAC ACC AGG GT | cluster 19A (um05302) | U. maydis |
| CH10 | TGA GGT GTC TTA GGA AGA GC |  | U. maydis |
| CH13 | TAG CAA GAT TCT CTG GAT CAC C | cluster 19A (um05294) | U. maydis |
| CH14 | CCG AGA GAG AAA ATG TAG GGA |  | U. maydis |
| CH15 | ATG AGA TCT CCC GCT CTT CTC | cluster 19A (um05295) | U. maydis |
| CH16 | TGT GAC AAA TCC GTG TAC TCT G |  | U. maydis |
| CH17 | TGA AGA TCC TTT CTC TCC TCC T | cluster 19A (um12302) | U. maydis |
| CH18 | CCG TCG GAA ACT TTG ACA TAG T |  | U. maydis |
| CH19 | ATG TTG AAA GTT CAA CCT CGG C | cluster 19A (um10553) | U. maydis |
| CH20 | GCT TGA CAC TAT TGA CGT CGG T |  | U. maydis |
| CH21 | ATG AAC CTT TCC TGT GTG CA | cluster 19A (um10554) | U. maydis |
| CH22 | AAA ACC GTG TAG CGT GAT TG |  | U. maydis |
| CH23 | ATG TGC TCG CAT CGG AAC A | cluster 19A (um05299) | U. maydis |
| CH24 | TCT TTG CCA GCA ACT CTT CC |  | U. maydis |
| CH25 | ATG CAC GCA ACC TTC CTC T | cluster 19A (um05300) | U. maydis |
| CH26 | CAG ATT AGG ACG ACG GAC TCC |  | U. maydis |
| CH27 | GCG TCA CAT CGC TCT TTC TCA T | cluster 19A (um05301) | U. maydis |
| CH28 | CAT AGA GCT CGA CGA TAG GAG G |  | U. maydis |
| CH29 | TCT AAC GAT CCT CGT ACT GTG G | cluster 19A (um05303) | U. maydis |
| CH30 | CCA GCG ATG TGA CCA AAG TTT A |  | U. maydis |
| CH31 | CAA TTT GTG CTT TGG TTA GCG C | cluster 19A (um10555) | U. maydis |
| CH32 | CCA TGC GAA CGA AAC CTT TCA A |  | U. maydis |
| CH33 | ATG CGC TCC AGC TTG ATT T | cluster 19A (um05305) | U. maydis |
| CH34 | TGA ATC TTT GAG GAT AGC CGT C |  | U. maydis |
| CH35 | ATG CAT TCC TCT GGC CTT G | cluster 19A (um05306) | U. maydis |
| CH36 | CTG GAT GGG AAT TGC AAT G |  | U. maydis |
| CH37 | CAA TGC TCA CTT TGC GGA G | cluster 19A (um05308) | U. maydis |
| CH38 | CAA TGC TCA CTT TGC GGA G |  | U. maydis |
| CH39 | CCT CTT GTT CAA AAC GCA TTG G | cluster 19A (um05309) | U. maydis |
| CH40 | GGG GCT CAT TTG AAG TAA GGT |  | U. maydis |
| CH41 | ACC TTG CTT CTG ATA GCT CTT G | cluster 19A (um05310) | U. maydis |
| CH42 | AGA GCC ACC CGA TCA CAT T |  | U. maydis |
| CH43 | ATG CAG CTC TGC GTC TCC A | cluster 19A (um05311) | U. maydis |
| CH44 | CGC TTT GCT CGA TCA GCT G |  | U. maydis |
| CH45 | ATG ACC TCG CTC CAT ATA CTG C | cluster 19A (um05312) | U. maydis |
| CH46 | TCC AAG AGG AAT CGC TTC G |  | U. maydis |
| CH47 | ATG GAC ACC AGC AAG TCT TGC | cluster 19A (um05313) | U. maydis |
| CH48 | CGA GGC AAC TCG TAA ATC GA |  | U. maydis |
| Cluster, Gene | Strain | Accession Numbers |  |
| CH49 | ATG TTG CAC AAG CTT CCA AC | cluster 19A (um05314) | U. maydis |
| CH50 | CGA GTG GTA CAA TCC TGT TCT C |  | U. maydis |
| CH51 | ATG ACG ATG ATC GGC AAG C | cluster 19A (um10557) | U. maydis |
| CH52 | TTG TAG GAT TCT CGC CAG AAG |  | U. maydis |
| CH53 | GCA AAA ACA AGC CCA GAG A | cluster 19A (um05316) | U. maydis |
| CH54 | CAG GCC CAA TGA AGT TGA TT |  | U. maydis |
| CH55 | ATG AAA GTT GGC TTG CCG G | cluster 19A (um05317) | U. maydis |
| CH56 | AAC AAC TCC GTT TGT CTG GTG |  | U. maydis |
| CH57 | CTA CCT CTT CAT CTC CTC TGG C | cluster 19A (um05318) | U. maydis |
| CH58 | TTC CGG AAA ATG AGC CTC G |  | U. maydis |
| CH59 | ATG CGT AAC TCA CGG TTC ATG | cluster 19A (um05319) | U. maydis |
| CH60 | GGT CGT CTC GAT CTC TTG GT |  | U. maydis |
| CH61 | GTT TGA CGA CTT TGT TCA GAG C | cluster 19A (um05292) | U. maydis |
| CH62 | AGC TTT GGT GGA GCA GTT G |  | U. maydis |
| CH63 | ATG CTG GAC GTC AAC ACT ATC G | cluster 19A (um05291) | U. maydis |
| CH64 | GCG AGT GCT TCG TAT TGC T |  | U. maydis |
| CH65 | ATG CGG GTC ACA CTC TTG A | cluster 19A (um05290) | U. maydis |
| CH66 | TCT GCT CCC ATC CAT CGA G |  | U. maydis |
| CH67 | ATC AAA GGA TCG AAA AGG TCG G | cluster 19A (um10705) | U. maydis |
| CH68 | GAC CAA AGT CGG CGA GTT T |  | U. maydis |
| CH69 | ATG ACA GTG CAA AAT GCT CCC | cluster 19A (um10552) | U. maydis |
| CH70 | CCA TCA GCA CTG AGT AGA TTC G |  | U. maydis |
| CH71 | CTT CGC TTC CTT ATA AAA CGG C | cluster 19A (um10559) | U. maydis |
| CH72 | CGA CTC GTC TAC AGA GCT AGC A |  | U. maydis |
| CH73 | ATG GTT ACG ACG TGC GAG T | cluster 19A (um05322) | U. maydis |
| CH74 | CGC ATA CTA GTC GAG CTT TCA |  | U. maydis |
| CH75 | GAT CAA GAT CCA ACC CAT CAA G | cluster 19A (um10560) | U. maydis |
| CH76 | AAC CAT TGC TGC GTC CTG A |  | U. maydis |
| CH77 | ATG TCT ACA ATA CCT CTC CGG C | cluster 19A (um10561) | U. maydis |
| CH78 | GTC CCA GTG CTT CTC ATC G |  | U. maydis |
| ITS1 | TCC GTA GGT GAA CCT GCG G | ITS |  |
| ITS4 | TCC TCC GCT TAT TGA TAT GC | ITS |  |
| 983F | GCY CCY GGH CAY CGT GAY TTY AT | ef1-α |  |
| 1567R | ACH GTR CCR ATA CCA CCR ATC TT | ef1-α |  |
| 2218R | ATG ACA CCR ACR GCR ACR GTY TG | ef1-α |  |
| G3PD-581F | CTC TTC CAT AAT GTC TCA GGT CAA C | gapdh | Ustilaginaceae |
| G3PD-2020R | CCT TTT AAC TTA CGC GCT GTC CTT C | gapdh | Ustilaginaceae |
| G3PD-1501R | GAA CTT GTC GTG GAT GAC CTT G | gapdh | Ustilaginaceae |
| RoK157 | AGG TGG CRC AGT GGA AYT GGA G | rpb1 | Ustilaginomycotina |
| RoK158 | CAC ATY GGY TAC CTY GGC AAG G | rpb1 | Ustilaginomycotina |
| RoK205 | CCA GCT ATT GAG CTC GTT TG | cluster 2A (um01233) | U. maydis |
| RoK206 | AGT CAG CGG ACA TGT TAA TC | cluster 2A (um01237) | U. maydis |
| RoK207 | GTC GCC TCA TCG TAG ATT TG | cluster 2A (um01237) | U. maydis |
| RoK208 | AGA ATG GTT TGG TCC TTT GG | cluster 2A (um01242) | U. maydis |
| RoK256 | AGA TCA ATC ACG GGC AGA AC | cluster 19A (um10553) | U. maydis |
| RoK257 | TCG CAA CCT TCA ACA ATG TC | cluster 19A (um10553) | U. maydis |
| Cluster, Gene | Strain | Accession Numbers |  |
| RoK258 | AGG GCA CGA TGA ACC TTT CC | cluster 19A (um10554) | U. maydis |
| RoK259 | GCA ACC GAG AGC CTA AAC AG | cluster 19A (um10554) | U. maydis |
| RoK317 | TGA TAT GCT GGC GAG CAG AG | pep1 (5´UTR) | U. maydis |
| RoK318 | GGT TTG CGA CGA CCA CAT AG | pep1 (3´UTR) | U. maydis |
