# Supplementary material for: Patterns of Variation at Ustilago maydis Virulence Clusters 2A and 19A Largely Reflect the Demographic History of Its Populations
Source: PLoS One. 2014 Jun 2;9(6):e98837. doi: 10.1371/journal.pone.0098837 (PMC4041787; doi:10.1371/journal.pone.0098837)
Supplement: Table S4 — Accession numbers. (DOC) [file pone.0098837.s006.doc]

Kellner et al. Table S4

| Cluster, Gene | Strain | Accession Numbers |
| --- | --- | --- |
| Gapdh (um02491) partial | RK001, RK020, RK122, RK123, RK124, RK126, RK127, RK134, RK139, RK208, RK209, RK212, RK213, RK214, RK215 | JF313635, JF313636, JF313637, JF313638,  JF313639, JF313640, JF313641, JF313642,  JF313643, JF313644, JF313645, JF313646,  JF313647, JF313648, JF313649 |
| Ef1-α (um00924) partial | RK001, RK020, RK042, RK122, RK123, RK124, RK126, RK127, RK134, RK139, RK208, RK209, RK212, RK213, RK214, RK215 | JF313619, JF313620, JF313621, JF313622,  JF313623, JF313624, JF313625, JF313626,  JF313627, JF313628, JF313629, JF313630,  JF313631, JF313632, JF313633, JF313634 |
| pep1 | RK001 | HQ002861 |
| pep1 | RK020 | HQ002862 |
| pep1 | RK042 | HQ002863 |
| pep1 | RK122 | HQ002864 |
| pep1 | RK123 | HQ002865 |
| pep1 | RK124 | HQ002866 |
| pep1 | RK126 | HQ002867 |
| pep1 | RK127 | HQ002868 |
| pep1 | RK134 | HQ002869 |
| pep1 | RK139 | HQ002870 |
| pep1 | RK208 | HQ002871 |
| pep1 | RK209 | HQ002856 |
| pep1 | RK212 | HQ002857 |
| pep1 | RK213 | HQ002858 |
| pep1 | RK214 | HQ002859 |
| pep1 | RK215 | HQ002860 |
| 2A | RK042 | HQ002872 |
| 2A | RK123 | HQ002873 |
| 2A | RK124 | HQ002874 |
| 2A | RK126 | HQ002875 |
| 2A | RK127 | HQ002876 |
| 2A | RK134 | HQ002877 |
| 2A | RK139 | HQ002878 |
| 2A | RK209 (FB2) | HQ002883 |
| 2A | RK212 | HQ002879 |
| 2A | RK213 | HQ002880 |
| 2A | RK214 | HQ002881 |
| 2A | RK215 | HQ002882 |
| 19A, um05290 (partial) | RK122, RK123, RK124, RK126, RK127, RK134, RK208, RK209, RK217 | HQ003208, HQ003209, HQ003210, HQ003211, HQ003212, HQ003213, HQ003214, HQ003215, HQ003216 |
| 19A, um05291 (partial) | RK122, RK123, RK124, RK126, RK127, RK134, RK208, RK209, RK217 | HQ003199, HQ003200, HQ003201, HQ003202, HQ003203, HQ003204, HQ003205, HQ003206, HQ003207 |
| 19A, um05292 (partial) | RK122, RK123, RK124, RK126, RK127, RK134, RK208, RK209, RK217 | HQ003190, HQ003191, HQ003192, HQ003193, HQ003194, HQ003195, HQ003196, HQ003197, HQ003198 |
| 19A, um05293 (partial) | RK122, RK123, RK124, RK126, RK127, RK134, RK208, RK209, RK217 | HQ003181, HQ003182, HQ003183, HQ003184, HQ003185, HQ003186, HQ003187, HQ003188, HQ003189 |
| 19A, um05294 (partial) | RK122, RK123, RK124, RK126, RK127, RK134, RK208, RK209, RK217 | HQ003172, HQ003173, HQ003174, HQ003175, HQ003176, HQ003177, HQ003178, HQ003179, HQ003180 |
| 19A, um05295 (partial) | RK122, RK123, RK124, RK126, RK127, RK134, RK208, RK209, RK217 | HQ003163, HQ003164, HQ003165, HQ003166, HQ003167, HQ003168, HQ003170, HQ003169, HQ003171 |
| 19A, um05299 (partial) | RK122, RK123, RK124, RK126, RK127, RK134, RK208, RK209, RK217 | HQ003154, HQ003155, HQ003156, HQ003157, HQ003158, HQ003159, HQ003160, HQ003161, HQ003162 |
| 19A, um05300 (partial) | RK122, RK123, RK124, RK126, RK127, RK134, RK208, RK209, RK217 | HQ003145, HQ003146, HQ003147, HQ003148, HQ003149, HQ003150, HQ003151, HQ003152, HQ003153 |
| 19A, um05302 (partial) | RK122, RK123, RK124, RK126, RK127, RK134, RK208, RK209, RK217 | HQ003131, HQ003128, HQ003129, HQ003132, HQ003133, HQ003130, HQ003134, HQ003135, HQ003127 |
| 19A, um05303 (partial) | RK122, RK123, RK124, RK126, RK127, RK134, RK208, RK209, RK217 | HQ003118, HQ003119, HQ003120, HQ003121, HQ003122, HQ003123, HQ003124, HQ003125, HQ003126 |
| 19A, um05305 (partial) | RK122, RK123, RK124, RK126, RK127, RK134, RK208, RK209, RK217 | HQ003113, HQ003115, HQ003116, HQ003117,  HQ003114, HQ003110, HQ003109, HQ003111, HQ003112 |
| Cluster, Gene | Strain | Accession Numbers |
| 19A, um05306 (partial) | RK122, RK123, RK124, RK126, RK127, RK134, RK208, RK209, RK217 | HQ003100, HQ003101, HQ003102, HQ003103, HQ003104, HQ003105, HQ003106, HQ003107, HQ003108 |
| 19A, um05308 (partial) | RK122, RK123, RK124, RK126, RK127, RK134, RK208, RK209, RK217 | HQ003091, HQ003092, HQ003093, HQ003094, HQ003095, HQ003096, HQ003097, HQ003098, HQ003099 |
| 19A, um05309 (partial) | RK122, RK123, RK124, RK126, RK127, RK134, RK208, RK209, RK217 | HQ003082, HQ003083, HQ003084, HQ003085, HQ003086, HQ003087, HQ003088, HQ003089, HQ003090 |
| 19A, um05310 (partial) | RK122, RK123, RK124, RK126, RK127, RK134, RK208, RK209, RK217 | HQ003073, HQ003074, HQ003075, HQ003076, HQ003077, HQ003078, HQ003079, HQ003080, HQ003081 |
| 19A, um05311 (partial) | RK122, RK123, RK124, RK126, RK127, RK134, RK208, RK209, RK217 | HQ003067, HQ003070, HQ003069, HQ003064, HQ003072, HQ003071, HQ003065, HQ003066, HQ003068 |
| 19A, um05312 (partial) | RK122, RK123, RK124, RK126, RK127, RK134, RK208, RK209, RK217 | HQ003055, HQ003056, HQ003057, HQ003058, HQ003059, HQ003060, HQ003061, HQ003062, HQ003063 |
| 19A, um05313 (partial) | RK122, RK123, RK124, RK126, RK127, RK134, RK208, RK209, RK217 | HQ003046, HQ003047, HQ003048, HQ003049, HQ003050, HQ003051, HQ003052, HQ003053, HQ003054 |
| 19A, um05314 (partial) | RK122, RK123, RK124, RK126, RK127, RK134, RK208, RK209, RK217 | HQ003037, HQ003038, HQ003039, HQ003040, HQ003041, HQ003042, HQ003043, HQ003044, HQ003045 |
| 19A, um05316 (partial) | RK122, RK123, RK124, RK126, RK127, RK134, RK208, RK209, RK217 | HQ003028, HQ003029, HQ003030, HQ003031, HQ003032, HQ003033, HQ003034, HQ003035, HQ003036 |
| 19A, um05317 (partial) | RK122, RK123, RK124, RK126, RK127, RK134, RK208, RK209, RK217 | HQ003019, HQ003020, HQ003024, HQ003022, HQ003021, HQ003025, HQ003023, HQ003026, HQ003027 |
| 19A, um05318 (partial) | RK122, RK123, RK124, RK126, RK127, RK134, RK208, RK209, RK217 | HQ003010, HQ003011, HQ003012, HQ003013, HQ003014, HQ003015, HQ003016, HQ003017, HQ003018 |
| 19A, um05319 (partial) | RK122, RK123, RK124, RK126, RK127, RK134, RK208, RK209, RK217 | HQ003001, HQ003002, HQ003003, HQ003004, HQ003005, HQ003006, HQ003007, HQ003008, HQ003009 |
| 19A, um05322 (partial) | RK122, RK123, RK124, RK126, RK127, RK134, RK208, RK209, RK217 | HQ002992, HQ002993, HQ002994, HQ002995, HQ002996, HQ002997, HQ002998, HQ002999, HQ003000 |
| 19A, um10552 (partial) | RK122, RK123, RK124, RK126, RK127, RK134, RK208, RK209, RK217 | HQ002983, HQ002984, HQ002985, HQ002986, HQ002987, HQ002988, HQ002989, HQ002990, HQ002991 |
| 19A, um10553 | RK122, RK123, RK124, RK126, RK127, RK134, RK208, RK209, RK217 | HQ002977, HQ002979, HQ002974, HQ002975, HQ002976, HQ002980, HQ002981, HQ002982, HQ002978 |
| 19A, um10554 | RK122, RK123, RK124, RK126, RK127, RK134, RK208, RK209, RK217 | HQ002965, HQ002966, HQ002967, HQ002968, HQ002969, HQ002970, HQ002971, HQ002972, HQ002973 |
| 19A, um10555 (partial) | RK122, RK123, RK124, RK126, RK127, RK134, RK208, RK209, RK217 | HQ002961, HQ002958, HQ002959, HQ002963, HQ002957, HQ002964, HQ002960, HQ002962, HQ002956 |
| 19A, um10556 (partial) | RK122, RK123, RK124, RK126, RK127, RK134, RK208, RK209, RK217 | HQ002947, HQ002948, HQ002949, HQ002950, HQ002951, HQ002952, HQ002953, HQ002954, HQ002955 |
| 19A, um10557 (partial) | RK122, RK123, RK124, RK126, RK127, RK134, RK208, RK209, RK217 | HQ002938, HQ002939, HQ002940, HQ002941, HQ002942, HQ002943, HQ002944, HQ002945, HQ002946 |
| 19A, um10558 (partial) | RK122, RK123, RK124, RK126, RK127, RK134, RK208, RK209, RK217 | HQ002936, HQ002932, HQ002934, HQ002933, HQ002937, HQ002935, HQ002930, HQ002929, HQ002931 |
| 19A, um10559 (partial) | RK122, RK123, RK124, RK126, RK127, RK134, RK208, RK209, RK217 | HQ002920, HQ002921, HQ002922, HQ002923, HQ002924, HQ002925, HQ002926, HQ002927, HQ002928 |
| 19A, um10560 (partial) | RK122, RK123, RK124, RK126, RK127, RK134, RK208, RK209, RK217 | HQ002911, HQ002912, HQ002913, HQ002914, HQ002915, HQ002916, HQ002917, HQ002918, HQ002919 |
| 19A, um10561 (partial) | RK122, RK123, RK124, RK126, RK127, RK134, RK208, RK209, RK217 | HQ002902, HQ002903, HQ002904, HQ002905, HQ002906, HQ002907, HQ002908, HQ002909, HQ002910 |
| 19A, um10705 (partial) | RK122, RK123, RK124, RK126, RK127, RK134, RK208, RK209, RK217 | HQ002901, HQ002898, HQ002894, HQ002895, HQ002896, HQ002899, HQ002897, HQ002893, HQ002900 |
| 19A, um12302 (partial) | RK122, RK123, RK124, RK126, RK127, RK134, RK208, RK209, RK217 | HQ002884, HQ002885, HQ002886, HQ002887, HQ002888, HQ002889, HQ002890, HQ002891, HQ002892 |
| 19A, um05301 (partial) | RK122, RK123, RK124, RK126, RK127, RK134, RK208, RK209, RK217 | HQ003136, HQ003137, HQ003138, HQ003139, HQ003140, HQ003141, HQ003142, HQ003143, HQ003144 |
