# Supplementary material for: Patterns of Variation at Ustilago maydis Virulence Clusters 2A and 19A Largely Reflect the Demographic History of Its Populations
Source: PLoS One. 2014 Jun 2;9(6):e98837. doi: 10.1371/journal.pone.0098837 (PMC4041787; doi:10.1371/journal.pone.0098837)
Supplement: Table S5 — Rates of Pa and Ps. Rates of Pa and Ps were calculated according to the number of non-synonymous and synonymous sites and non-synonymous and synonymous mutations per locus. stdv: standard deviation. (DOC) [file pone.0098837.s007.doc]

Kellner et al. Table S5

|  | Pa | Pa stdv | Ps | Ps stdv | Pa/Ps | Locus | protein prediction |
| --- | --- | --- | --- | --- | --- | --- | --- |
| um01987 | 0,0044 | 0,0023 | 0,01501 | 0,0073 | 0,29 | pep1 | secreted |
| um00924 | 0,0003 | 0,0003 | 0,00201 | 0,0014 | 0,15 | ef1*-α* | non-secreted |
| um01238 | 0,0037 | 0,0015 | 0,0021 | 0,0012 | 1,76 | 2a | secreted |
| um01235 | 0,0022 | 0,0008 | 0,0014 | 0,00101 | 1,57 | 2a | secreted |
| um01237 | 0,00201 | 0,0007 | 0,0025 | 0,0012 | 0,80 | 2a | secreted |
| um01234 | 0,0028 | 0,0011 | 0,00501 | 0,0024 | 0,56 | 2a | secreted |
| um01239 | 0,00401 | 0,0017 | 0,0088 | 0,0045 | 0,46 | 2a | secreted |
| um01240 | 0,0011 | 0,0008 | 0,00301 | 0,0024 | 0,37 | 2a | secreted |
| um01236 | 0,0011 | 0,0006 | 0,0061 | 0,0026 | 0,18 | 2a | secreted |
| um01242 | 0,0004 | 0,0003 | 0,00401 | 0,0017 | 0,10 | 2a | secreted |
| um01233 | 0,00 | 0,00 | 0,0035 | 0,0022 | 0,00 | 2a | non-secreted |
| um10553 | 0,00401 | 0,0019 | 0,0044 | 0,0035 | 0,91 | 19a | secreted |
| um10554 | 0,0034 | 0,0018 | 0,0038 | 0,0028 | 0,89 | 19a | secreted |
| um05301 | 0,0045 | 0,0019 | 0,0051 | 0,0029 | 0,88 | 19a | secreted |
| um05294 | 0,0039 | 0,0024 | 0,0072 | 0,0051 | 0,54 | 19a | secreted |
| um05300 | 0,0022 | 0,0017 | 0,0054 | 0,0054 | 0,41 | 19a | secreted |
| um05299 | 0,0021 | 0,0016 | 0,0065 | 0,0051 | 0,32 | 19a | secreted |
| um05303 | 0,0037 | 0,0027 | 0,0154 | 0,0091 | 0,24 | 19a | secreted |
| um05302 | 0,00101 | 0,0007 | 0,0048 | 0,0049 | 0,21 | 19a | secreted |
| um05310 | 0,0007 | 0,0007 | 0,0041 | 0,0029 | 0,17 | 19a | secreted |
| um12302 | 0,0019 | 0,0019 | 0,01301 | 0,0092 | 0,15 | 19a | secreted |
| um10556 exon | 0,0005 | 0,0005 | 0,0057 | 0,0033 | 0,09 | 19a | secreted |
| um10559 | 0,0035 | 0,0025 | 0,00401 | 0,00401 | 0,87 | 19a | non-secreted |
| um05313 exon | 0,0018 | 0,0018 | 0,00501 | 0,00501 | 0,36 | 19a | non-secreted |
| um05292 | 0,0023 | 0,0018 | 0,0096 | 0,0068 | 0,24 | 19a | non-secreted |
| um10560 | 0,0018 | 0,0018 | 0,0131 | 0,0093 | 0,14 | 19a | non-secreted |
| um05290 | 0,0018 | 0,0019 | 0,01701 | 0,01001 | 0,11 | 19a | non-secreted |
